# Zebrafish Adjust Their Behavior in Response to an Interactive Robotic Predator

**DOI:** 10.3389/frobt.2019.00038

**Published:** 2019-05-31

**Authors:** Chiara Spinello, Yanpeng Yang, Simone Macrì, Maurizio Porfiri

**Affiliations:** ^1^Department of Mechanical and Aerospace Engineering, New York University, Tandon School of Engineering, Brooklyn, NY, United States; ^2^Key Laboratory of Mechanism Theory and Equipment Design of Ministry of Education, School of Mechanical Engineering, Tianjin University, Tianjin, China; ^3^Centre for Behavioural Sciences and Mental Health, Istituto Superiore di Sanità, Rome, Italy; ^4^Department of Biomedical Engineering, New York University, Tandon School of Engineering, Brooklyn, NY, United States

**Keywords:** *Danio rerio*, ethorobotics, fear, interactive robots, transfer entropy

## Abstract

Zebrafish (*Danio rerio*) constitutes a valuable experimental species for the study of the biological determinants of emotional responses, such as fear and anxiety. Fear-related test paradigms traditionally entail the interaction between focal subjects and live predators, which may show inconsistent behavior throughout the experiment. To address this technical challenge, robotic stimuli are now frequently integrated in behavioral studies, yielding repeatable, customizable, and controllable experimental conditions. While most of the research has focused on open-loop control where robotic stimuli are preprogrammed to execute a priori known actions, recent work has explored the possibility of two-way interactions between robotic stimuli and live subjects. Here, we demonstrate a “closed-loop control” system to investigate fear response of zebrafish in which the response of the robotic stimulus is determined in real-time through a finite-state Markov chain constructed from independent observations on the interactions between zebrafish and their predator. Specifically, we designed a 3D-printed robotic replica of the zebrafish allopatric predator red tiger Oscar fish (*Astronotus ocellatus*), instrumented to interact in real-time with live subjects. We investigated the role of closed-loop control in modulating fear response in zebrafish through the analysis of the focal fish ethogram and the information-theoretic quantification of the interaction between the subject and the replica. Our results indicate that closed-loop control elicits consistent fear response in zebrafish and that zebrafish quickly adjust their behavior to avoid the predator's attacks. The augmented degree of interactivity afforded by the Markov-chain-dependent actuation of the replica constitutes a fundamental advancement in the study of animal-robot interactions and offers a new means for the development of experimental paradigms to study fear.

## Introduction

Zebrafish (*Danio rerio*) has recently emerged as a relevant experimental species for the study of functional and dysfunctional biological processes (Stewart and Kalueff, 2012; Stewart et al., 2012). The use of zebrafish in biomedical research rests upon a series of advantages, including their high homology to mammals' physiology, short intergeneration time, high reproduction rate, and external fertilization, along with the availability of a sequenced genome and the possibility to stock them at a higher density compared to laboratory rodents (Kalueff et al., 2014a). Kalueff et al. outlined the validity of zebrafish as an experimental model for the study of executive functions and emotional responses (Kalueff et al., 2012). Within this framework, zebrafish have been used to investigate the mechanisms underlying higher order brain functions, such as learning and memory (Al-Imari and Gerlai, 2008; Pather and Gerlai, 2009), or the exhibition of emotional patterns (Maximino et al., 2010; Steenbergen et al., 2011), like fear and anxiety (Stewart et al., 2012).

Zebrafish are sensitive to a wide range of experimental stressors. For example, previous studies reported that novelty-exposure (Cachat et al., 2010) alarm substances (Speedie and Gerlai, 2008), and predator exposure (Gerlai, 2013) may trigger anxiety and fear responses in zebrafish. The presence of live predators has been repeatedly reported to induce fear response in zebrafish, thereby prompting the integration of sympatric and allopatric predators in a number of behavioral paradigms. For example, the visual exposure to Indian leaf fish (*Nandus nandus*), a sympatric predator of zebrafish, resulted in increased bottom dwelling (geotaxis), shoal cohesion, and predator avoidance (Stewart et al., 2014). In a binary-choice test, we showed that zebrafish exhibit robust aversion towards an allopatric predator, the red tiger Oscar fish *(Astronotus ocellatus)* (Ladu et al., 2015).

While the use of live predators may beget relevant information regarding the fundamental mechanisms of emotional responses, it nonetheless presents a major limitation. The use of live predators as independent variables is flawed by the fact that they may not always show consistent behaviors, thereby failing to guarantee full controllability to the experimenters. Specifically, live predators can exhibit inconsistent behavioral patterns across experimental trials, due to tiredness and potential idiosyncrasies with focal subjects, and their behavior may fluctuate following physiological variations. The use of computerized images constitutes a valid alternative to compensate for the lack of controllability of live stimuli (Gerlai et al., 2009; Luca and Gerlai, 2012; Gerlai, 2013). However, computer-animated images cannot successfully reproduce the three-dimensional complexity of a live predator (Woo and Rieucau, 2011). While size and morphology of a live stimulus can be adequately mimicked in computer animated images, other features, like depth, motion, and texture, cannot be equivalently represented. For example, depth cues, as reported in Woo and Rieucau (2011), provide information about the distance between the object and other elements in the surrounding environment.

Limitations of live predators and computer-animated images might be overcome through the use of biologically-inspired robots (Krause et al., 2011; Butail et al., 2015; Porfiri, 2018; Romano et al., 2018). In previous work published by our group, we showed that zebrafish exposed to a robot inspired by a sympatric predator, the Indian Leaf Fish, exhibited robust antipredatorial aversion in a binary-choice test (Cianca et al., 2013). In a more recent effort, we reported that zebrafish exhibited aversion for a robotic replica that mimicked the morphology and the tail beating of an allopatric predator, the red tiger Oscar fish (Ladu et al., 2015). Moreover, we reported that, differently from the robotic replica, computer animated images of the red tiger Oscar fish did not elicit aversion in zebrafish, supporting the idea that computerized images may not reproduce the complexity of a live predator (Woo and Rieucau, 2011; Ladu et al., 2015).

While these studies highlighted the possibility of modulating animal behavior through customizable and controllable stimuli, they did not allow for interactivity in the response of the robot. Specifically, most of these studies were performed in open-loop control, where the robot was programmed to perform an a-priori chosen behavior or follow a known trajectory without responding to the behavior of the live subject (Polverino et al., 2012; Bonnet et al., 2014; Ruberto et al., 2016; Cazenille et al., 2017; Bierbach et al., 2018a,b). To bridge this gap, recent efforts have involved a closed-loop control system, in which the motion patterns of robotic stimuli are contingent upon the behavior of live fish (Swain et al., 2012; Kopman et al., 2013; Landgraf et al., 2013, 2016; Bonnet et al., 2018; Kim et al., 2018).

For example, Swain et al. (2012) introduced a cyber-physical system that enabled a robotic replica of a koi to use real-time feedback to control its movements in response to live fish. In particular, computer vision techniques were used to acquire the position of golden shiners and to create a real-time feedback for the predator's replica to attack the fish. The replica was magnetically connected to a wheeled robot that was positioned underneath the tank and maneuvered the robotic koi (Swain et al., 2012). Using a similar setup, Landgraf et al. (2013, 2016) investigated social interactions in guppies (*Poecilia reticulata*) and in the three-spined stickleback (*Gasterosteus aculeatus*).

More recently, some research groups developed closed-loop control systems to study collective behavior in zebrafish. For example, Bonnet et al. (2018) developed a framework to perform experiments with mixed groups of live fish and robots in which the robots interacted in closed-loop with the zebrafish. Small wheeled mobile robots were used to magnetically maneuver fish lures in a circular corridor. The lures mimicked the morphology of zebrafish and passively beat their tails. The authors formed mixed groups of six individuals composed by three live zebrafish and three lures. Notably, in one of the experimental conditions, the robots were controlled to move in the direction of the group majority, thereby implementing a closed-loop control system.

Our previous efforts focused on closed-loop control in a binary-choice test (Kopman et al., 2013; Kim et al., 2018). In Kopman et al. (2013), the tail beat frequency of a stationary robot mimicking the aspect of a zebrafish conspecific was modulated according to the position of a focal zebrafish in the tank. The robot was positioned in one of the lateral compartments of a tri-partitioned tank while the focal subject was allowed to swim in the central compartment. More recently, in Kim et al. (2018), we examined closed-loop control in three dimensions. In particular, a zebrafish replica was maneuvered via a robotic arm capable of moving the replica along three dimensions and of minimizing the distance between the replica and the focal fish depending on zebrafish position in the tank.

Here, we propose an interactive robotics-based platform to study zebrafish fear response to a predator-like replica. Compared to previous efforts, the platform presented in this manuscript offers several technological and theoretical advancements, ranging from the field of study (fear-related response) to the adoption of an innovative mathematical framework (finite-state Markov chain to actuate the replica). We establish a closed-loop control system through the integration of 3D-printing and real-time computer vision tracking. In this system, a 3D-printed replica of an allopatric predator, the red tiger Oscar fish, is maneuvered by a robotic arm, based on real-time measurement of fish position. A custom-made software allows real-time tracking of the position of the focal subject in the experimental tank in three dimensions, by fusing two orthogonal camera views. The behavior of live red tiger Oscar fish has been visually scored to devise a finite state Markov chain, which was used to actuate the robotic predator across three different locomotory patterns, in response to the relative position of the live subject in the tank. Within this framework, the level of aggressiveness of the replica increased when the fish was in its proximity.

We used the red tiger Oscar fish instead of the sympatric predator Indian Leaf Fish for two reasons. First, previous studies reported that the red tiger Oscar fish elicit fear reactions in zebrafish (Oliveira et al., 2013; Ladu et al., 2015). Second, our choice rested upon the consideration that we wanted to avoid ceiling effects and allow a certain degree of variability in the behavior of the focal fish. Compared to the red tiger Oscar, the Indian Leaf Fish elicits a much stronger fear reaction [see also Bass and Gerlai (2008) and Cachat et al. (2011)]. Since in this study we tested the same replica exhibiting a differential motion pattern (open- and closed-loop), we wanted to limit the possibility to observe a ceiling effect (that is, fish exhibiting a maximal avoidance regardless of replica's motion pattern) that might have masked differential responses to the experimental conditions.

The robotic platform was tested on zebrafish in a set of binary choice experiments in which fish were systematically presented with the biologically-inspired replica of the predator with different levels of interactivity (open- or closed-loop). During the test, subjects and robots were separated by a transparent wall, allowing only visual interaction. Fear response of focal subjects was investigated by considering geotaxis (average distance between the fish and the tank's base, time spent in the bottom section of the tank, and number of entries into the bottom section of the tank) and avoidance (average distance from the replica and average percentage of time spent by the focal fish in the half of the water column opposite to that occupied by the replica). Additionally, we evaluated fish behavior in terms of speed, magnitude of the acceleration, and magnitude of the turn rate.

We hypothesized that the behavioral response of the focal subject would vary depending on the level of interactivity of the replica. In particular, we predicted an increase of avoidance and geotaxis for closed-loop control, where the replica would respond to the focal subjects' behavior. This hypothesis rests upon available evidence. In particular, several studies showed that live (Kalueff et al., 2014a; Stewart et al., 2014) and artificial predators (Gerlai et al., 2000, 2009; Cianca et al., 2013; Ladu et al., 2015) induced fear-related phenotypes in zebrafish. Geotaxis is a typical indicator of fear-related response. Zebrafish show geotaxis in response to the exposure to alarm pheromones (Cachat et al., 2011), to a novel (potentially dangerous) environment (Levin et al., 2007), and to predators (Stewart et al., 2014). Moreover, several efforts reported that the administration of anxiolytic drugs, such as ethanol (Stewart et al., 2011) and diazepam (Bencan et al., 2009), reduces geotaxis in zebrafish. To evaluate whether the degree of biomimicry of the replica was associated with variations in fear-related responses, we also evaluated the time spent attacking by the replica in open- or closed-loop. Had zebrafish successfully adapted to the closed-loop condition, the number of attacks received by the robot would be predicted to diminish over time due to the exhibition of an acquired avoidance to the robot.

Finally, we implemented the information-theoretic framework of transfer entropy (Schreiber, 2000) to further investigate the avoidance response of the live fish for the robotic predator. Transfer entropy allows to assess the strength and direction of the interaction between two dynamical systems from their raw time series. It offers a quantitative measurement of potential cause and-effect relationships between the two systems in a Wiener-Granger sense, such that a system is “caused” by the other if it is possible to reduce the uncertainty about its future prediction by using knowledge about the other system. Several studies demonstrated that transfer entropy constitutes a valid approach to investigate animal-robot interactions and predator-prey interactions (Butail et al., 2014; Hu et al., 2015; Orange and Abaid, 2015; Neri et al., 2017; Kim et al., 2018). With respect to this information-theoretic approach, we predicted that transfer entropy would help detect an information flow from the replica to the focal subject; in particular, we hypothesized that the state of the robot would affect the position of the fish. Such a prediction is in line with the hypothesis that the replica should induce an avoidance response in zebrafish.

## Materials and Methods

### Ethics Statements

Experiments were performed in accordance with relevant guidelines and regulations and were approved by the University Animal Welfare Committee (UAWC) of New York University under protocol number 13-1424.

### Animal Housing

A total of 48 wild-type zebrafish were purchased from Carolina Biological Supply Co. (Burlington, NC, USA) with a female/male ratio equal to 1:1. Upon arrival, fish average body length (BL) was around 3 cm. Fish were housed in five 37.5 L (10 gallons) vivarium tanks (Pentair Aquatic Eco-systems Locations, Cary, NC, USA) with a density of no more than 10 fish per tank. Prior to the beginning of the experiments, fish were acclimatized for at least 14 days in the holding facility. During habituation, males and females were kept separated within their housing tanks to facilitate sex identification during experimental sessions. Water parameters were regularly checked, and temperature and pH were maintained at 26°C and 7.2, respectively. Fish were kept in a 12 h light/12 h dark photoperiod (Cahill, 1996) and fed once a day with commercial flake food (Hagen Corp. Nutrafin max) around 7 PM.

### Interactive Robotic Platform

**Figure 1:**
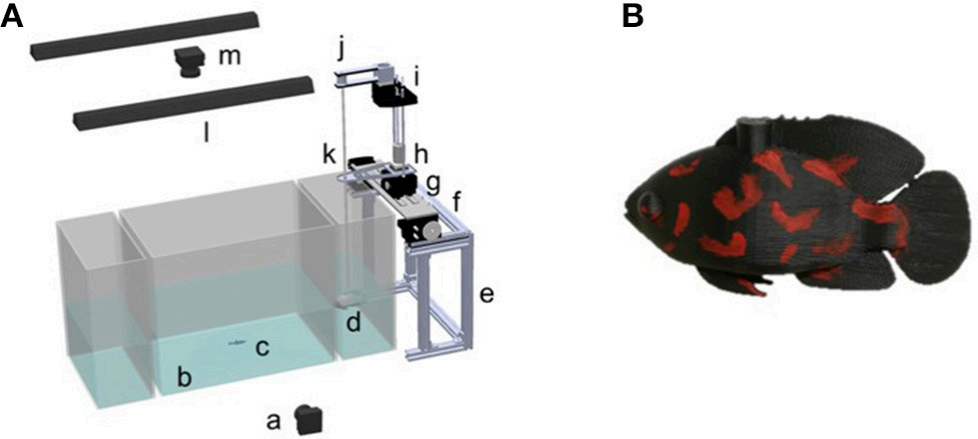
Schematic of the experimental apparatus with **(A)** the robotic platform, and **(B)** the red tiger Oscar fish replica. With respect to the platform, we identify **a**. The webcam used to capture the vertical plane of the experiment **b**. The experimental tank **c**. The live subject **d**. The replica **e**. The aluminum frame **f**. The servo-motor utilized for forward and backward movement **g**. The DC motor employed for the side movement **h**. The stepper motor used for the vertical motion of robot **i**. The stepper motor for heading control **j**. The pulley **k**. The auxiliary support to reduce the sway of the rod **l**. The light **m**. The webcam used to capture the top view of the experiment.

The platform used in this work builds upon our previous work (Ruberto et al., 2016; Kim et al., 2018). The frame of the platform was built from aluminum T-slot bars (McMaster Carr, Elmhurst, IL) (Figure 1). It affords four degrees of freedom in three dimensions. The movement along the *x*-axis (length of the tank) was achieved using two servo motors (HS-755HB, Hitec RCD, Poway, CA, USA) connected to a rack-and-pinions-gear (Robotzone, Winfield, KS, USA). Another rack-and-pinions gear (Robotzone, Winfield, KS, USA) was connected to a DC motor with an encoder (Robotzone, LLC, Winfield, Kansas, USA). Such a DC motor was utilized to maneuver the robot along the *y*-axis (width of the tank). Along the *z*-axis (height of the tank), a stepper motor (NEMA-17, Adafruit, New York City, New York, USA) was employed to actuate the motions of the replica via a threaded rod (McMaster Carr, Elmhurst, Illinois, USA). To control the heading of the robot, we used another stepper motor (NEMA-17, Adafruit, New York City, New York, USA), fixed on a cantilever and connected to a pulley set.

The replica was mounted on a transparent acrylic rod (HIC Technology Co., Ltd, China). An Arduino Uno microcontroller (Arduino, Italy) covered by a motor shield (Adafruit, New York, New York, USA) and an Ethernet shield (Arduino, Italy) was used to: (i) control the stepper motors for the movements along the *y*- and *z*-axes; (ii) control the servo motor for the movement along the *x*-axis; and (iii) communicate with a PC via a router. Another Arduino Uno microcontroller (Arduino, Italy) covered by another motor shield (Adafruit, New York, New York, USA) was utilized to control the heading of the replica.

A red tiger Oscar fish replica was designed in SolidWorks (Dassault Systèmes SolidWorks Corp., Waltham, Massachusetts, USA) and 3D-printed using a Dimension Elite. The replica was painted using non-toxic waterproof paint (Krylon, Krylon Products Group, Cleveland, Ohio, USA), to resemble a live red tiger Oscar predator.

A real-time tracking software, programmed in C++ language, was implemented in Visual Studio, 2015 (Microsoft, Redmond, WA, USA), using the open source computer vision library OpenCV v3.169 (Intel Corp., Santa Clara, CA, USA). The software enabled image acquisition from two orthogonal cameras and real-time tracking of a live fish in three dimensions. Specifically, at each time step, the frames were transformed into gray-scale images and smoothed by image blurring with an averaging window of 7 × 7 pixels to remove noise. Moving targets were detected by subtraction of two consecutive frames, and implementation of a binary filter, a dilation filter, and an eroding filter. After image processing, a blob detection algorithm was implemented to identify the centroid of the fish. If tracking was lost at any frame, the software implemented an adaptive search of potential blobs by changing the size of the searching region based on predictions about the position through a Kalman filter. If multiple objects were tracked, the new target was selected as the blob at the smallest distance from the last position tracked. To balance the distortion associated to the perspective view from each camera, two dimensional positions data from the top and front views were linearly interpolated and calibrated according to the dimensions of the tank, see Kim et al. (2018) for details.

### Apparatus

The experimental apparatus consisted of three transparent Plexiglas tanks of two different dimensions, see Figure 1. The larger tank, measuring 42 × 30 × 30 cm (length, width, and height), was placed between two smaller tanks with dimensions of 16 × 30 × 30 cm (length, width, and height). The tanks were placed within a frame of aluminum T-slot bars and were surrounded by black curtains to prevent visual disturbance from the environment. Additionally, the lateral side of each smaller tank and the bottom of all the tanks were covered with white contact paper to minimize disturbance from the outside and facilitate tracking. Two soft white curtains hung by transparent fish line (Berkley Trilene XT Extra Tough, Pure Fishing, Inc., Columbia, SC, USA) were placed between the central tank and the two smaller lateral tanks, to avoid visual contact between experimental fish and the robotic replica during the habituation time. Two 25 W fluorescent tubes (All-Glass Aquarium, UK) were mounted at 71 cm from the water surface to illuminate the experimental apparatus.

Two webcams (Logitech C920 webcam, Lausanne, Switzerland) were used to record the live fish and the robotic stimulus at 30 frames per second with a resolution of 640 × 480 pixels. One camera was positioned above the tank at a distance of 1.06 m and was used to capture the horizontal movements of the zebrafish and the robotic stimulus. The other camera was mounted on a frame perpendicularly to the front panel of the tank at a distance of 0.9 m and was used to capture the vertical motions of the fish and the replica.

### Open- and Closed-Loop Control

In this work, we utilized a finite-state Markov chain to control the motion of the replica. A finite Markov chain is a sequence of random variables (*X*_0_, *X*_1_….) within a finite state-space Ω which satisfies the following Markov property,

$$\begin{array}{l} \text{Pr}(X_{t+1}=x_{t+1}|X_{t}=x_{t}\ldots ,X_{0}=x_{0})\\ \;=\mathrm{Pr}(x_{t+1}=x_{t+1}|X_{t}=x_{t}) \end{array}$$

In the equation above, lowercase quantities denote realizations in Ω, and *t* is the discrete time step. In a sequence of states, the probability of a future state will rest upon only the current state and does not depend on the past (Brémaud, 2013). On the basis of observations of live predators reported in our pilot experiments (El Khoury et al., 2018), we constructed the finite state space from combinatorial collection of states of both the red tiger Oscar fish and the focal subject.

**Figure 2:**
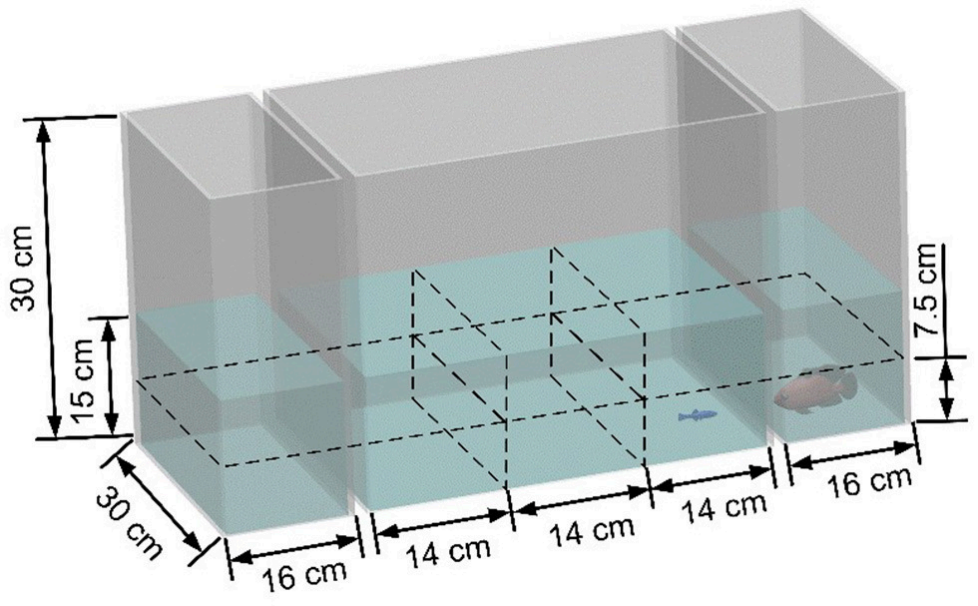
Schematic view of the experimental tank section used to determine the state of the experimental subject. The zebrafish and the robotic predator are considered in the swimming-close state (Sw-C) since the predator is swimming and the zebrafish is close to it.

The robotic replica switched among different states, termed “stationary,” “swimming,” and “attacking.” In the stationary state the replica was held fixed in place; in the swimming state it aimlessly moved around the tank; and in the attacking state it exhibited aggressive behavior in the form of trashing against the short side of the tank. The distance between the replica and the zebrafish was defined “far” or “close,” based on their relative position, discretized as follows. The length of the central tank was divided into three equal parts (quantiles), while the water column was divided in two sections, “upper” and “lower.” The fish was considered close to the replica if it was swimming in the quantile nearest to it and in its same section (lower or upper). Otherwise, the fish was considered to be far from the replica. For example, Figure 2 shows the swimming-close state. We considered the distance between the zebrafish and the predator's behavior to identify six states that classify predator-prey interaction: stationary-close (St-C), stationary-far (St-F), swimming-close (Sw-C), swimming-far (Sw-F), attacking-close (A-C), and attacking-far (A-F).

Similar to our pilot experiment (El Khoury et al., 2018), we built a transition matrix by observing videos in which the live predator and the zebrafish were interacting. In particular, ten 10-min long videos were scored by two independent observers that inspected the states of the predators. Such states were qualitatively defined on the basis of the following ethogram: (i) “stationary,” where the predator remained fixed in a place, with a complete cessation of movement (except for gills and eyes), for 4 s; (ii) “swimming,” where the predator moved aimlessly around the tank; and (iii) “attacking,” where the predator moved repeatedly back and forth along the tank's wall adjacent to the compartment where the live zebrafish was placed. Differently from our pilot experiment, we maximized the time spent attacking by the replica through the following procedure. For each video, the observers manually scored the occurrence of an attack every second, so that they assigned a one to each second in which they observed an attack and a zero when they observed swimming or stationary states. We aggregated the 10 videos into a single time series, in which at every second, we counted the fraction of videos reporting an attack. From this time series, we identified the 1-min long segment that featured the largest total fraction of attacks, and we calibrated the six by six transition matrix of the Markov chain on it.

In closed-loop control, the probability transition matrix M_CL_ was calculated as

(1) $$\begin{array}{l} {\text{M}}_{\text{CL}}=\\ \begin{array}{ll} & \begin{array}{llllll} \text{St}-\text{C} & \text{ St}-\text{F} & \text{Sw}-\text{C} & \text{Sw}-\text{F} & \text{A}-\text{C} & \text{A}-\text{F} \end{array}\\ \begin{array}{l} \text{St}-\text{C}\\ \text{St}-\text{F}\\ \text{Sw}-\text{C}\\ \text{Sw}-\text{F}\\ \text{A}-\text{C}\\ \text{A}-\text{F} \end{array} & \left[\begin{array}{llllll} 0.806 & 0.194 & 0 & 0 & 0 & 0\\ 0.075 & 0.891 & 0.007 & 0.027 & 0 & 0\\ 0.011 & 0.023 & 0.794 & 0.138 & 0.034 & 0\\ 0 & 0.016 & 0.060 & 0.891 & 0 & 0.033\\ 0 & 0 & 0.042 & 0 & 0.750 & 0.208\\ 0 & 0 & 0.043 & 0.043 & 0.203 & 0.711 \end{array}\right] \end{array} \end{array}$$

The closed-loop transition matrix was used in real-time to maneuver the robotic replica as a function of the position of the focal subject. Specifically, given a state for the robotic replica and the fish among the six possible options, the behavior of the robot was chosen based on the corresponding transition probabilities in the matrix. For example, if at a given time the robotic replica and the fish are in state Sw-C, we refer to the third row in the transition matrix M_CL_ to dictate the subsequent behavior of the robotic replica among its three possible states St, Sw, and A. With probability 0.011 + 0.023 = 0.034 the robot will become stationary (St-C or St-F), with probability 0.794 + 0.138 = 0.932 it will continue swimming (Sw-C or Sw-F), and with probability 0.034 it will attack (A).

The stationary distribution associated with this Markov chain, computed as the left eigenvector with unitary eigenvalue, is characterized by the following six probabilities, ordered as the six-dimensional state vector π_CL_: 0.110 (St-C), 0.265 (St-F), 0.134 (Sw-C), 0.277 (Sw-F), 0.106 (A-C), and 0.108 (A-F). By examining the distribution of the robotic replica's behavior as a function of the relative position of the fish, we find that if the fish is close to the replica there is roughly the same probability of the robot exhibiting any of the three behaviors (St: 0.314; Sw: 0.383; and A: 0.303), while being away will favor swimming and stationary states over the attacking state (St: 0.408; Sw: 0.426; and A: 0.166). For example, the numerical value 0.314 for the probability of the replica being stationary when the fish is close is obtained as π (St-C)/[π(St-C)+ π(Sw-C)+ π(A-C)].

In open-loop control, the state of the robot changed independently of the position of the zebrafish in the tank. The stationary distribution of the replica was obtained by simply marginalizing π_CL_ over the position of the fish, to determine the following three-dimensional vector, π_OL_: 0.375 (St), 0.412 (Sw), and 0.214 (A). The transition matrix was similarly computed, albeit with some extra steps, by marginalizing the closed-loop model in equation (1) over the state of the focal fish. Ultimately, we obtained the three-state Markov chain for the robot with states St, Sw, and A, with probability transition matrix M_OL_.

(2) $$\begin{array}{ll} & \begin{array}{lll} \text{St} & \text{Sw} & \text{A} \end{array}\\ \begin{array}{lll} & & \text{St}\\ {\text{M}}_{\text{OL}}=\text{Sw}\\ & & \text{A} \end{array} & \left[\begin{array}{lll} 0.976 & 0.024 & 0\\ 0.022 & 0.945 & 0.033\\ 0 & 0.064 & 0.936 \end{array}\right] \end{array}$$

For example, the entry corresponding to the probability of maintaining a stationary state in between two consecutive times, M_OL_(St, St), was derived from the matrix M_CL_ in equation (1), by marginalizing over the state of the fish and using the definition of conditional probability, such that one needed to aggregate the entries in the first two-by-two block of M_CL_ and weigh with respect to the stationary distributions. In formulas: M_OL_(St,St) = [M_CL_(St-C,St-C) + M_CL_(St-C,St-F)]π_CL_(St-C)/π_OL_(St) + [M_CL_(St-F,St-C) + M_CL_(St-F,St-F)]π_CL_(St-F)/π_OL_(St).

### Movement of the Stimulus

Similar to El Khoury et al. (2018), during the stationary state the replica was programmed to move vertically downwards to the bottom of tank and keep freezing until a transition to a new state was required. When the robot was in the swimming state, it moved along an elliptical trajectory in the horizontal plane with axes of lengths equal to 2.35 and 10 cm, selected based on the visual scoring of pilot trials. The nominal speeds along the *x*- and *y*-axis were 1.01 cm/s and 1.33 cm/s. To add randomness to the motion, for each occurrence of a swimming state, the speed along the *y*-axis was increased or decreased of 0.1 cm/s with a probability of 0.1. In the vertical plane, the robot would randomly ascend or dive for 1 cm with a probability of 0.2. Finally, the attacking motion consisted in a repeated movement back and forth, following the lateral wall next to the central tank.

### Experimental Conditions

Three experimental conditions were considered in this study. In the control condition, the platform was actuated without the replica attached to the transparent acrylic cantilever (see Supplementary Videos 1 and 2). Thus, the fish was allowed to see the rod moving in the lateral tank and to perceive the associated noise from the motors onboard the platform. In the open-loop condition (see Supplementary Videos 3 and 4), 16 sets of simulated state transitions were created to perform 16 trials. Each 20-min simulation, contained a sequence of 1,200 events, beginning with the stationary state. In the closed-loop condition (see Supplementary Videos 5 and 6), the replica was actuated as a function of the relative distance to the fish, acquired through the real-time tracking system.

### Experimental Procedure

Experiments were conducted between October and November 2018. Up to 10 trials were performed per day, for a maximum of five trials in the morning (between 10 a.m. and 1 p.m.) and five trials in the afternoon (between 2 and 6:30 p.m.) for a total of 16 trials per condition. Each trial was recorded using real-time tracking software for 16 min, including 10 min of habituation, and 6 min of observation. At the beginning of the experiments, the robotic platform was placed in one of the two lateral tanks. One experimentally naïve fish was randomly chosen from different holding tanks and gently hand netted in the central tank inside the experimental apparatus. The same number of naïve fish was maintained in each vivarium tanks throughout the experiments. Trials were randomized to balance sex of the experimental fish, lateral tank (left or right), and the time of the day (morning or afternoon). During habituation, the lateral side of the central tank was covered with a white curtain in order to prevent the fish to see the replica inside the lateral tank. After 10 min, the curtains were manually removed (using fish lines from above the setup), to allow the visual perception of the stimulus during the observation time. After the experiments, the fish was placed back in the vivarium and kept separated from the naïve fish. Each fish was used only once.

### Data Analysis

The raw data collected by the tracking system included the position of live fish and robot in space and the states of the robot.

Consistent with the implementation of the closed-loop control system, the avoidance for the robotic replica was evaluated using two different parameters: (i) the average distance between the fish and the replica, calculated from the tracked position of the fish and the tracked position of the replica (for open- and closed-loop conditions) or the geometric center of the water volume in the lateral tank (for the control condition); and (ii) the time spent by the focal fish in the half of the water column opposite to that occupied by the replica. The latter parameter was computed by dividing the water column in two ideal sections of equal height, that is, upper and lower sections. The percentage of time spent by the fish in the lower section when the replica was positioned in the upper one was added to the percentage of time spent by the fish in the upper section when the replica was positioned in the lower one. This parameter required the computation of the time spent by the robotic replica in the lower half of the water column.

Fish geotaxis was evaluated through the computation of three different parameters: (i) the average distance between the fish and the base of the tank; (ii) the average time spent in the bottom of the tank (defined as the bottom third of the water column); and (iii) the number of entries into the bottom section of the tank.

Fish activity was estimated by measuring three different parameters: average speed, average magnitude of the acceleration, and average magnitude of the turn rate. The speed of the fish was computed via a first-order numerical differentiation of the trajectory data. Similarly, the acceleration was computed based on a first-order numerical differentiation of the velocity data. The magnitude of the turn rate, ω_*t*_, was computed through the following equation:

(3) $$\begin{array}{l} \omega_{t}=\frac{1}{△t}\cos^{-1}\frac{v_{t}\;\cdot v_{t+1}}{||v_{t}||\;||v_{t+1}||} \end{array}$$

where *v*_*t*_ and *v*_*t*+1_ are the velocity vectors at time step *t* and *t*+1, and △*t* is the duration of a time step (1/30 s). The acquired position data were smoothed using a moving average with a window size of 18 frames to reduce the noise in the velocity computation, used in the speed and magnitude of the turn rate. A similar procedure was executed on the velocity data to estimate the acceleration.

Statistical analyses were performed using R 3.5.0. The linear mixed-effects model with “condition” (control, open-loop or closed-loop) and “time” (minutes) as fixed factors and the unique identity of each fish as random factor has been performed. Model comparison was performed using the “ANOVA” function from the base package (Speekenbrink and Konstantinidis, 2015; Wenger et al., 2016). Statistical significance level was chosen at 0.05. When significance was registered, *post-hoc* analysis were performed using “glht” function (Hothorn et al., 2008) for multiple comparisons.

To study the interaction between robot and fish, we computed transfer entropy from the state of replica to the position of fish, measured along the width or the depth of the tank. Transfer entropy (TE) from the replica (*R*) to the fish (*F*) was computed as Schreiber (2000),

(4) $${\text{TE}}_{R\to F}=\sum_{F_{t+1,\;F_{t},\;R_{t}}}P(F_{t+1},F_{t},R_{t})\log_{2}\frac{P(F_{t+1}|F_{t},R_{t})}{(F_{t+1}|F_{t})}$$

where *P* is the probability mass function, estimated from the time series. Based on our previous work (Porfiri, 2018), we binned fish position with a resolution of 1 BL (3 cm) and we down-sampled the data at 1 Hz. These selections mitigate the need of delays or memory effects in the transfer entropy computation, while resulting in time series of about 600 data points that could support robust inference of probability mass functions. *R*_*t*_ is the state of the robot at time step *t*, taking three possible values: attacking, swimming, and stationary. *F*_*t*_ is the binned position of the fish along the length (14 bins) or the depth (5 bins) of the tank. For each of the two experimental conditions (open- and closed-loop) and for each of the selected fish position (longitudinal and vertical), we computed 16 values of TE_*R*→*F*_ from the available 16 pairs of time series.

To estimate significance of transfer entropy results, we compared the value of transfer entropy to surrogate data obtained by shuffling the time series. Specifically, for each experimental condition and for each choice of the fish position, we shuffled the dataset so that the 16 time series for the fish were randomly paired with the 16 time series of the states of robotic replica. For each permutation of the 16 pairs, we computed a mean value of transfer entropy and we repeated this process 1,000 times to generate a distribution for surrogate mean transfer entropy. We ascertained significance of transfer entropy results by checking whether the mean value of TE_*R*→*F*_ was located in the right tail (≥95%) of the distribution of the surrogated data.

## Results

### Avoidance

**Figure 3:**
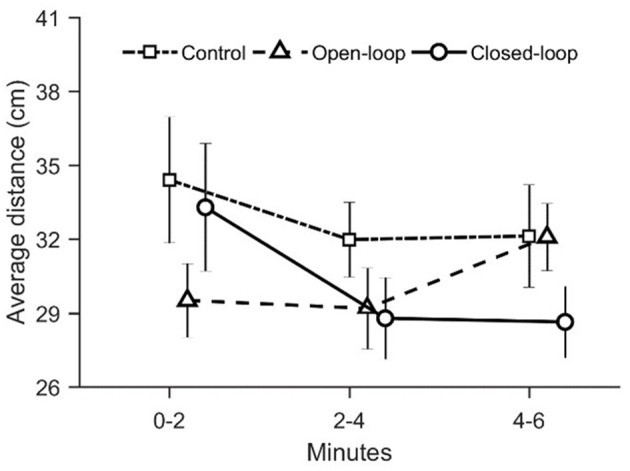
Average distance over time between the live fish and the replica (in open- and closed-loop conditions), or between the live fish and the center of the replica's tank (in the control condition). Data are reported as mean ± standard error of the mean.

**Figure 4:**
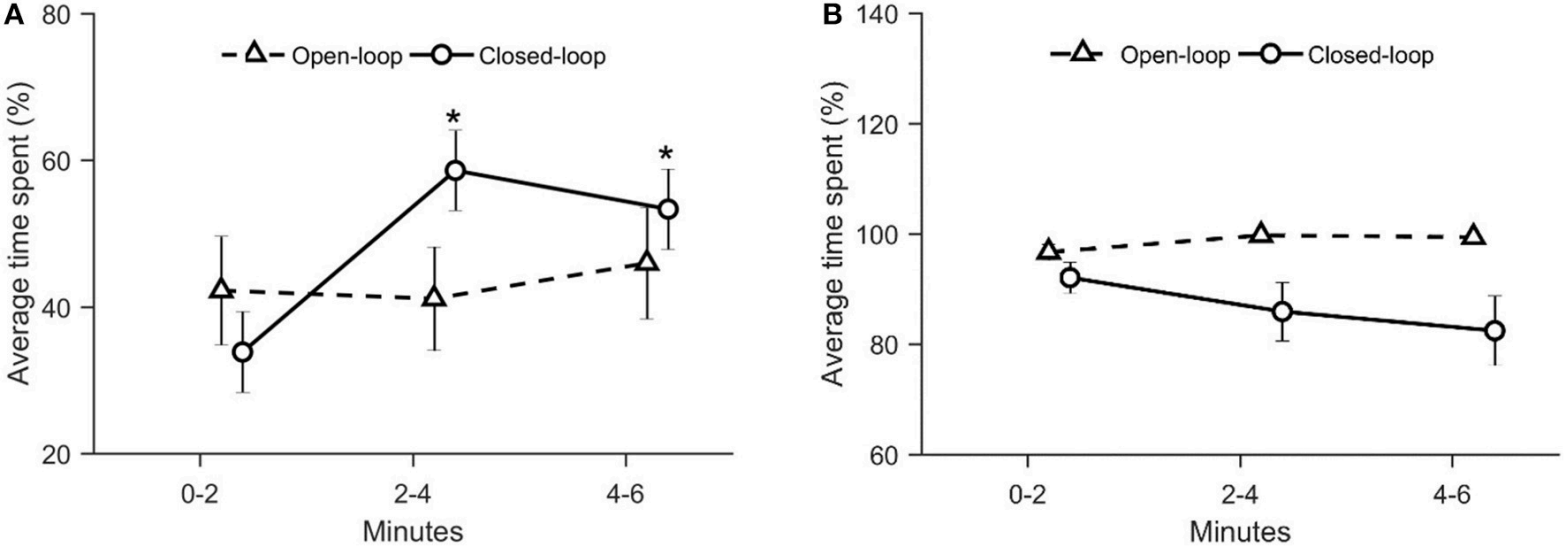
**(A)** Average time spent by the focal fish in the half of the water column opposite to that occupied by the robotic replica. The water column has been divided in two ideal sections of equal height (upper and lower). The percentage of time spent by the fish in the lower section when the replica was positioned in the upper section was added to the percentage of time spent by the fish in the upper section when the replica was positioned in the lower. An asterisk indicates a significant difference in *post-hoc* comparisons (*p* < 0.010) with the first-time bin (0–2 min) within the same experimental group. Data are reported as mean ± standard error of the mean. **(B)** Average percentage of time spent by the robot in the lower half of the water column. The latter was obtained by dividing the water column in two ideal sections of equal height (upper and lower). Data is reported as mean ± standard error of the mean.

To evaluate zebrafish fear response to the robotic replica, we computed two avoidance-related parameters: the average distance between the replica and the fish and the time spent by the focal fish in the half of the water column opposite to that occupied by the replica. While experimental groups did not differ in terms of average distance from the replica [condition: $\chi_{\left(2\right)}^{2}$ = 1.80, *p* = 0.41; time: $\chi_{\left(2\right)}^{2}$ = 2.93, *p* = 0.23; see Figure 3], they exhibited a differential time-dependent profile with respect to the time spent in the section of the water column opposite to that occupied by the replica [condition × time: $\chi_{\left(2\right)}^{2}$ = 11.4, *p* = 0.003; see Figure 4A].

This metric did not vary over time in fish tested in the open-loop condition; conversely, fish tested in the closed-loop condition exhibited a considerable increase in this metric during the second (min 2–4) and third fraction of the experiment (min 4–6), compared to the first two experimental minutes (*p* < 0.010 in *post-hoc* comparisons; see Figure 4A). Since this variable was a function of the position of the replica, we also quantified the time spent by the replica in the lower half of the water column. We observed that such a parameter was significantly higher in open-loop than in closed-loop [condition: $\chi_{\left(1\right)}^{2}$ = 11.1, *p* < 0.01; see Figure 4B]. Yet, it did not vary over time in either open- and closed-loop conditions [time: $\chi_{\left(2\right)}^{2}$ = 1.09, *p* = 0.58; see Figure 4B], thereby suggesting that the behavior of the focal fish varied despite the fact that the position of the replica remained constant throughout the entire experiment.

**Figure 5:**
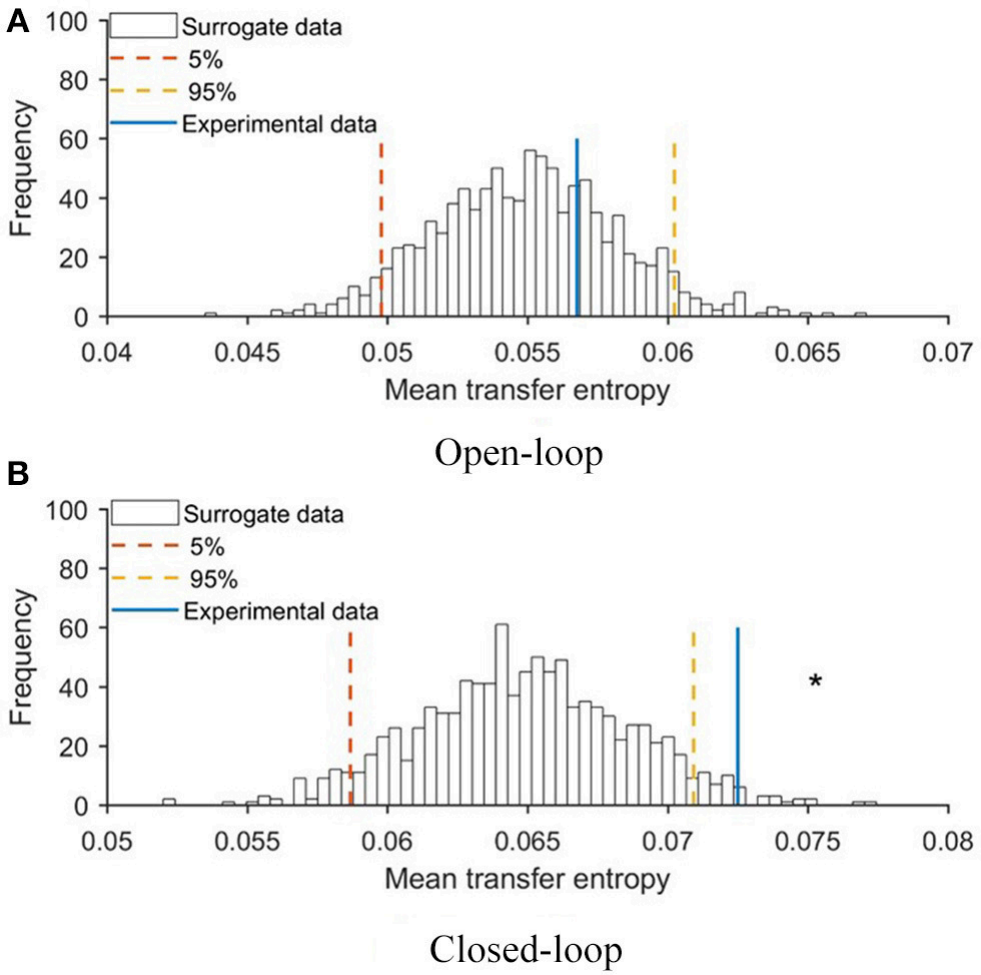
Mean values of transfer entropy from the state of the robot to the vertical position of the fish compared with surrogate data set for **(A)** open- and **(B)** closed-loop conditions. Red and yellow lines represent 5 and 95% quantile of the probability distributions, respectively. An asterisk represents a significant (*p* < 0.050) difference of transfer entropy from chance.

**Figure 6:**
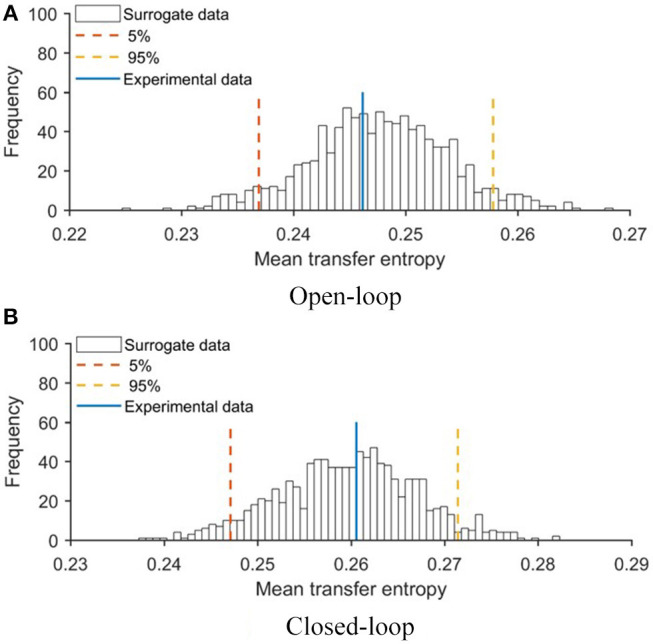
Mean values of transfer entropy from the state of the robot to the horizontal position of the fish compared with surrogate data set for **(A)** open- and **(B)** closed-loop conditions. Red and yellow lines represent 5 and 95% quantile of the probability distributions, respectively.

To evaluate the extent to which the robotic replica influenced the behavior of the focal subjects, we quantified transfer entropy from the robot to the fish in closed- and open-loop conditions. Transfer entropy bestows a direct measure of the improved ability to infer the future state of the focal subject from its current one, due to additional knowledge about the present state of the robot. To statistically substantiate the significance of this analysis, we first generated a probability distribution of transfer entropy values through a bootstrapping approach (see Materials and Methods, section Data Analysis) and then compared real values obtained in closed- and open-loop conditions with this probability distribution. In Figure 5, we report the probability distribution of mean transfer entropy (black histograms), highlight the 5% and 95% quantile (dashed lines), and the actual value of the mean transfer entropy observed in each condition (full line). Values above the right dashed line indicate a significant information transfer, that is, experimental conditions in which the motion of the replica significantly influenced the behavior of the experimental subject. This analysis showed that the state of the robot affected the vertical position of the fish in the closed-loop condition (see Figure 5B) and not in the open-loop condition (see Figure 5A). Additionally, transfer entropy analysis indicates that the state of the robot does not affect the position of the fish along the horizontal axis of the tank neither in open-loop (see Figure 6A) nor in closed-loop (see Figure 6B).

### Geotaxis

Geotaxis was computed as the average height in the water column, the average time spent in the bottom of the tank, and the average number of entries into the bottom section of the tank.

**Figure 7:**
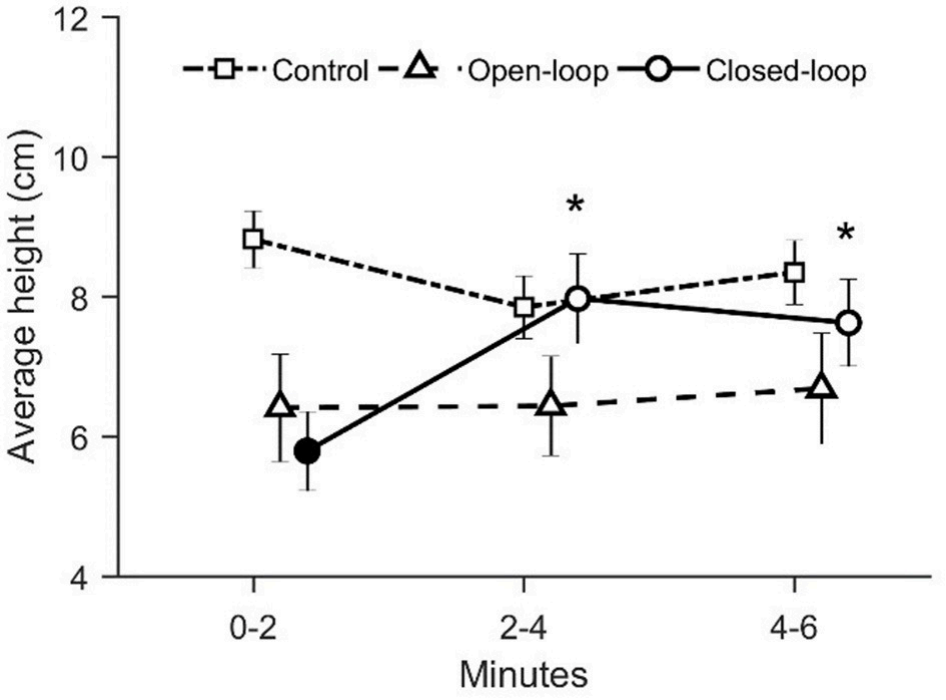
Average distance between the fish and the base of the tank. The asterisk indicates a significant difference in *post-hoc* comparisons (*p* < 0.010) with the first-time bin (0–2 min) within the same experimental group. The full symbol represents a significant difference in *post-hoc* comparisons (*p* < 0.010) with control group during the first-time bin (0–2 min). Data are reported as mean ± standard error of the mean.

With respect to the average height in the water column, we identified a significant time-dependent variation across conditions [condition × time: $\chi_{\left(4\right)}^{2}$ = 18.7, *p* = 0.001; see Figure 7]. In particular, during the first two experimental minutes, fish tested in the closed-loop condition were characterized by a reduced average height along the water column compared to the control condition (*p* < 0.010 in *post-hoc* comparison; see Figure 7). Additionally, we observed a significant increase of the average height in the closed-loop condition after the first-time interval (min 0–2) (*p* < 0.010 in *post-hoc* comparison; see Figure 7).

**Figure 8:**
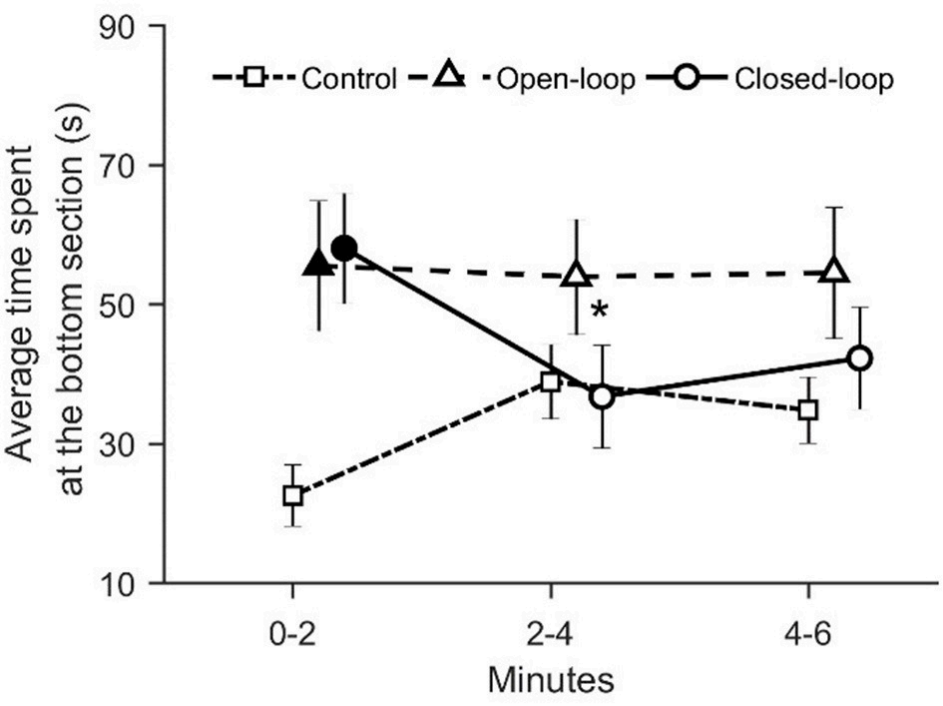
Time spent at the bottom of the tank (corresponding to the lower 5 cm). The asterisk indicates a significant difference in *post-hoc* comparisons (*p* < 0.050) with the first-time bin (0–2 min) within the same experimental group. Full symbols represent a significant difference in *post-hoc* comparisons (*p* < 0.050) with control group during the first time bin (0–2 min). Data are reported as mean ± standard error of the mean.

We registered that visual exposure to the robotic replica significantly increased the time spent in the bottom section of the water column throughout the experimental session [condition × time: $\chi_{\left(4\right)}^{2}$ = 15.8, *p* = 0.003; see Figure 8]. During the first two experimental minutes, fish tested in open- and closed-loop conditions spent more time in the bottom section compared to subjects in the control condition (*p* < 0.050 and *p* < 0.010, respectively in *post-hoc* comparisons; see Figure 8). Additionally, in the closed-loop condition, we observed a significant reduction in the time spent at the bottom of the tank during the second part of the experiment (min 2–4), compared to the first two experimental minutes (*p* < 0.050 in *post-hoc* comparison; see Figure 8).

**Figure 9:**
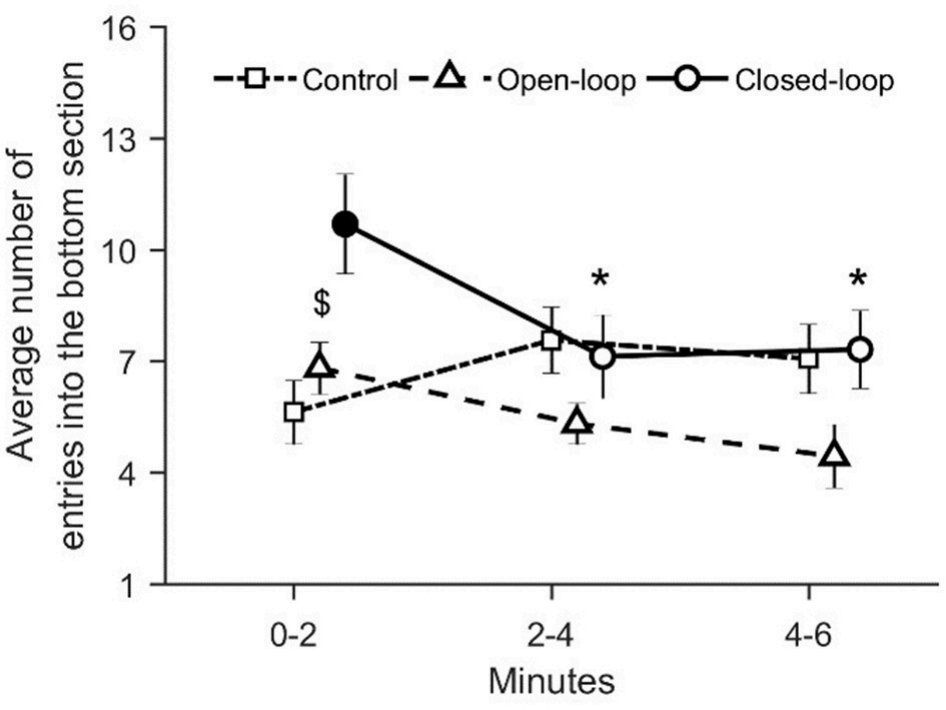
Average number of entries into the bottom section. The asterisk indicates a significant difference in *post-hoc* comparisons (*p* < 0.010) with the first time bin (0–2 min) within the same experimental group. The full symbol represents significance in *post-hoc* comparisons (*p* < 0.010) with control group at the first-time step (0–2 min). Data are reported as mean ± standard error of the mean.

With respect to the average number of entries in the bottom section of the tank, we registered a significant variation in closed-loop over time [condition × time: $\chi_{\left(4\right)}^{2}$ = 22.4, *p* = 0.001; see Figure 9]. In particular, we noted that during the first two experimental minutes, closed-loop interactions resulted in a significant increase in the number of entries in the bottom section of the tank compared to control and open-loop conditions (*p* < 0.010 and *p* < 0.050, respectively, in *post-hoc* comparisons; see Figure 6). Additionally, fish tested in the closed-loop condition exhibited a significantly lower number of entries during the second and the third time intervals (min 2–4 and 4–6), compared to the first 2 min (*p* < 0.010 and *p* < 0.010 in *post-hoc* comparisons; see Figure 9).

**Figure 10:**
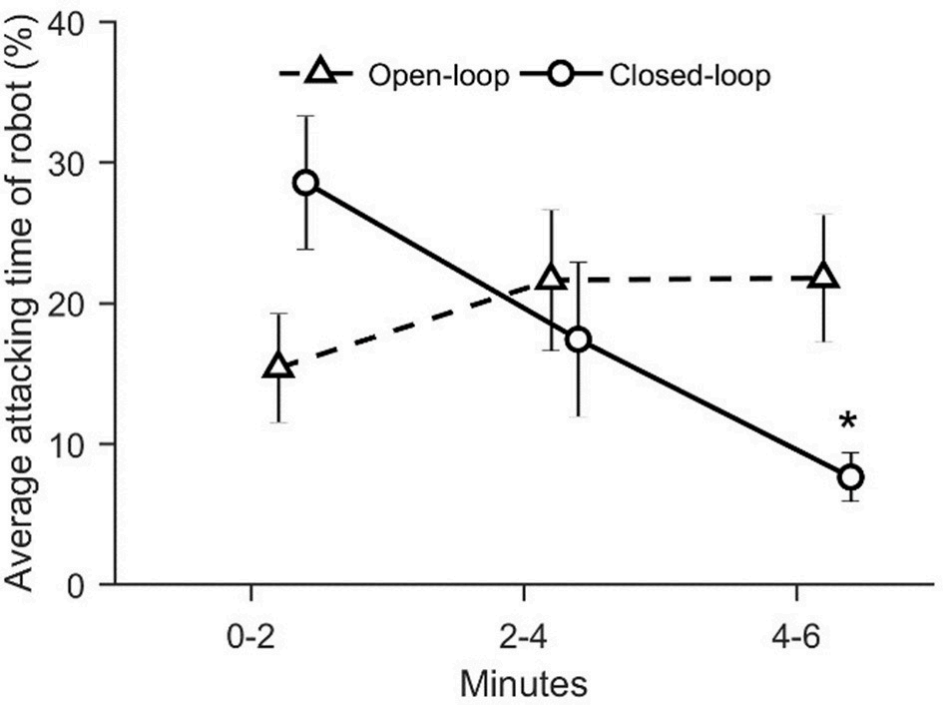
The average percentage value of attacking state of robot. The asterisk indicates a significance in *post-hoc* comparisons (*p* < 0.010) with the first-time step (0–2 min) within the same experimental group. Data is reported as mean ± standard error of the mean.

With the aim of evaluating zebrafish behavioral response as a function of the degree of the replica's interactivity, we computed the average percentage of time spent attacking by the replica and the percentage of time spent by the replica in the lower half of the water column. We detected a significant variation in closed-loop condition in the time spent attacking over the six experimental minutes [condition × time: $\chi_{\left(2\right)}^{2}$ = 12.1, *p* = 0.016; see Figure 10]. In particular, the time spent attacking by the closed-loop robot significantly decreased during the third interval of the experiment compared to the first 2 min (*p* < 0.010 in *post-hoc* comparison; see Figure 10).

### Fish Activity

To evaluate fish activity, we computed the average speed, average magnitude of the acceleration, and average magnitude of the turn rate. While we did not observe differences among conditions in the average speed [condition: $\chi_{\left(2\right)}^{2}$ = 3.09, *p* = 0.213; see Table 1], all the experimental groups showed a time-dependent decrease in the average speed [$\chi_{\left(2\right)}^{2}$ = 0.094, *p* < 0.001; see Table 1]. We registered a significant reduction of the fish average magnitude of the acceleration in open-loop condition [condition: $\chi_{\left(2\right)}^{2}$ = 7.45, *p* = 0.024; see Table 1]. In particular, we detected a significant decrease in open-loop compared to the control condition (*p* < 0.010 in *post-hoc* comparison). Additionally, the average magnitude of the acceleration significantly decreased over-time in all experimental groups [time: $\chi_{\left(2\right)}^{2}$ = 25.0, *p* < 0.001; see Table 1]. Finally, with respect to the average magnitude of the turn rate, we did not identify significant differences among experimental groups or over time [condition: $\chi_{\left(2\right)}^{2}$ = 0.343, *p* = 0.843: time: $\chi_{\left(2\right)}^{2}$ = 2.65, *p* = 0.266; see Table 1].

**Table 1:** Fish activity: average speed, average magnitude of the acceleration, and average magnitude of the turn rate.

	**Control**	**Open-loop**	**Closed-loop**	**Condition**		**Time**		**Condition × time**	
				**$\chi_{\left(2\right)}^{2}$**	***p***	**$\chi_{\left(2\right)}^{2}$**	***p***	**$\chi_{\left(2\right)}^{2}$**	***p***
Average speed (cm/s)	7.24 ± 0.130	6.24 ± 0.080	7.02 ± 0.106	3.09	0.213	19.6	0.0006	6.58	0.160
Average magnitude of the acceleration (cm/s^2^)	13.1 ± 0.797	9.67 ± 0.345	11.8 ± 0.402	7.45	0.024	25.0	<0.001	6.86	0.143
Average magnitude of the turn rate (rad/s)	3.61 ± 0.059	3.511 ± 0.057	3.713 ± 0.071	0.340	0.843	2.65	0.266	15.3	0.053

*Mean values and standard error means. The three rightmost columns indicate χ^2^ value and p-value of the main effect of condition, time, and their interaction*.

## Discussion

Here, we studied the interactions between live zebrafish and a biologically-inspired replica of an allopatric predator, the red tiger Oscar fish, using a robotics-based platform. The replica was actuated by a robotic arm along four degrees of freedom represented by movements along three independent axes and control of body oscillations. Interactive experiments were implemented through a custom-made real-time tracking software that allowed the measurement of the position of a live zebrafish in the experimental tank. In particular, the motion of the replica along the three axes was controlled from the real-time tracked position of the fish, ultimately causing the replica to respond as a function of the fish ethogram. We performed three experimental conditions aimed at quantifying how the degree of interactivity of the robot affects the behavioral response of live zebrafish. We conducted the experiments in a canonical binary-choice test, where fish were allowed to swim in the central tank while the replica was actuated in a lateral tank. The replica and the live subject were separated by a transparent Plexiglas panel that allowed only visual interactions. Fish behavioral response was studied through the integration of classical anxiety-related parameters (avoidance, geotaxis, and activity) and an information-theoretic approach that allows to disentangle the cause-and-effect relationships at the base of the interaction between the replica and the fish.

In accordance with our predictions, during the first two experimental minutes, the robotic replica elicited fear-related response in zebrafish, in terms of geotaxis. In particular, we observed that during the early stages of test fish spent more time at the bottom section of the tank compared to controls in both open- and closed-loop conditions. Geotaxis is a typical indicator of fear-related response in zebrafish (Kalueff et al., 2013): diving toward the bottom of the tank is generally considered an anti-predatorial avoidance response. This result confirms previous observations according to which the visual exposure to a predatorial stimulus elicits fear-related behavior in zebrafish (Kalueff et al., 2014a). Additionally, we noted that the strength of the geotaxis response increased with the degree of the replica's interactivity. In particular, we registered a lower average distance from the base of the tank in the closed-loop condition compared to control. Concerning the comparison between open-loop and control condition, although data inspection suggested the presence of a difference between these two groups during the first two minutes of testing, such a difference was not statistically significant. Considering the number of entries into the bottom level, we observed an increase in the closed-loop condition compared to both open-loop and control conditions. On the contrary, the open-loop condition did not differ from the control condition.

We suggest that the elevated geotaxis observed during the first two experimental minutes in the closed-loop condition may depend on the increased instances of the replica mimicking an attack. Differently from the open-loop condition, the closed-loop robot was programmed to adjust its locomotion patterns based on the relative position of the fish in the tank, such that the probability of occurrence of an attack was higher if the fish was close to the replica. We reported that the average distance between the replica and the experimental fish did not vary among conditions during the entire experimental session. At the same time, during the first two minutes, the time spent by the focal fish in the half of the water column opposite to that occupied by the replica did not differ between open- and closed-loop conditions. Additionally, even though the two percentages are not significantly different, the time spent attacking by the closed-loop robot (~30%) during the first two minutes of the experiment is remarkably higher than the time spent attacking by the open-loop robot (~15%). Thus, the higher interactivity of the closed-loop robot resulted in a higher time spent attacking during the first two experimental minutes which, in turn, manifested in increased geotaxis.

Our results showed a time-dependent reduction in geotaxis depending on the increase of interactivity of the robot. In closed-loop, we observed a significant increase of the average distance from the tank's base and a significant decrease in both the average time spent and the number of entries into the bottom section after the first two experimental minutes. We suggest that fish tested in closed-loop might have adjusted their behavior to minimize predator's attacks. This hypothesis rests upon the fact that, after the first two minutes, we observed an increase of avoidance for the robotic replica in closed-loop condition. In particular, fish tested in closed-loop spent more time in the section opposite to the one occupied by the robot. Such a behavior resulted in a significant reduction in attacks simulated by the robot during the last two experimental minutes. It is tenable to propose that the elevated time spent attacking by the closed-loop robot induced, after the first two minutes, the fish to move to the higher part of the water column to avoid the predator.

Our explanation is supported by previous findings (Cachat et al., 2011). In particular, Cachat et al. reported that zebrafish displayed shorter latency to enter the upper half of the tank and more time spent in the upper half when visually exposed to their live sympatric predator, the Indian leaf fish (Cachat et al., 2011). The authors explained such a difference reporting that the predator spent most of its time at the bottom of the tank; consequently zebrafish might have learned to move toward the upper part of the tank to avoid the predator (Cachat et al., 2011). We may suggest that during the first two minutes of the experiment fish tested in closed-loop condition reacted to the replica through geotaxis. Then, given the high time spent by the replica attacking, they moved toward the upper part of the tank to avoid the robotic predator. Ultimately, we propose that the behavioral responses exhibited by closed-loop subjects throughout the experimental session may reflect two different antipredatorial strategies: an early strategy characterized by a sudden preference for the bottom of the experimental tank and a later one characterized by more complex behaviors aimed at minimizing the number of attacks received.

In partial disagreement with our intuition and with previous efforts, zebrafish visual exposure to the replica did not manifest into a significant increase in average distance from the replica (a classical parameter of avoidance). Cachat et al. reported that the visual exposure to a live Indian leaf fish induces avoidance and manifestation of erratic movements in zebrafish (Cachat et al., 2011). The same erratic movements were induced by the visual exposure to a live red tiger Oscar fish (Cachat et al., 2011). Similarly, in our previous work, we reported that the visual exposure to a robotic replica of red tiger Oscar fish elicits aversion in a binary choice test and increasing of thrashing behavior (Ladu et al., 2015). We may suggest that, differently from Ladu et al. (2015), the three-dimensional motion of the replica might have offered an alternative strategy to avoid the replica. While in our previous work the replica was fixed in the middle of the water column with just the tail beating, here the replica was maneuvered in three dimensions and its position in the water column and, more in general, in the lateral tank, varied. Thus, fish might have developed a different strategy to avoid the replica, that is, moving toward the upper part of the tank after the first two minutes from the beginning of the experimental session. As already outlined, a reduced latency to move toward the upper part of the tank and an increased time spent at the top part of the tank have been reported as a measure of predator's avoidance by Cachat et al. (2011).

The possibility that the behavior of the fish was directly influenced by the robot is supported by transfer entropy data. Specifically, building on our previous work (Porfiri, 2018) and related studies (Bossomaier et al., 2016; Moore et al., 2018), we used transfer entropy to infer cause and-effect relationships between the live fish and the robotic replica. Our results confirm that fish adjusted their behavior as a function of the degree of interactivity of the robot. Specifically, we observed that the vertical motion of the fish was influenced by the state of the robotic replica in closed-loop control. A similar response was not identified when measuring the horizontal position, in agreement with the absence of a significant effect of the robot on the average distance between the replica and the fish.

The theoretical advantages of the use of robotic stimuli are represented by their reproducibility, their customizability and hence by their higher degree of controllability (compared to live stimuli) throughout the entire experimental session. As shown in our previous work, the behavior shown by a live zebrafish, when confronted with a robot, is less variable compared with that exhibited in response to a live stimulus, be the latter a conspecific (Spinello et al., 2013) or a predator (Cianca et al., 2013; Ladu et al., 2015). Here, we tested a robotic platform capable of actuating three different states, swimming, stationary and attacking, inspired by the motion of a live predator. For the first time in a robotics-based platform, we integrated the complex interplay between predator and prey through the introduction of closed-loop control implemented via a finite-state Markov chain. Although a similar platform has been considered in our previous study (Kim et al., 2018), the present work begets several innovations. Rather than focusing on preference toward zebrafish-inspired replicas, we adopted a closed-loop control system to investigate fear response toward a predator-like replica. Based on this experimental design, we replaced the simple following behavior of the replica with a richer repertoire. The latter was achieved by implementing a finite-state Markov chain formulated from real-life interaction between zebrafish and their allopatric predator red tiger Oscar.

Beyond addressing practical limitations of the platform highlighted in Kim et al. (2018), future efforts should seek to afford physical contact between the fish and replica and improve the degree of biomimicry of the replica. In fact, the presence of the Plexiglas walls did not allow physical interactions between the stimulus and the robotic predator. Such a physical barrier might be perceived as a protection for the live fish and might have mitigated the avoidance response to the predator. Future studies should allow physical interaction between the replica and the focal subject while maintaining the closed-loop control system. With respect to the biomimicry of the robotic replica, we identified two specific issues that could be improved in future research, that is, the replica's ethogram and its body undulations. Toward the aim of reproducing predator's behavior, we considered three different states: swimming, stationary and attacking. The latter consisted of a motion where the robotic stimulus moved back and forth along the *x*-axis of the tank near the transparent wall. However, as reported in Beeching (1997), frontal display, charge, and bites are also recognized as attack activities in cichlids. Future studies should aim at enriching the behavioral repertoire of the robotic replica. At the same time, the material utilized to print the replica does not allow to reproduce the flexibility of the live predator's body. Future work should explore the possibility of printing the replica in a more flexible material, like silicone. Finally, another line of potential inquiry could explore the complex interplay between fear response induced by the robotic replica and social behavior, to shed some light on the strategies that are used by groups to avoid predators.

In conclusion, this study puts forward an interactive-based approach to study fear response in zebrafish induced by an interactive robotic predator in three dimensions. We expect that this robotic tool will be utilized in translational study involving zebrafish. For example, the platform will be useful to investigate the mechanisms underlying physiological and pathological processes related to emotional domains, both in baseline conditions and in response to psychoactive compounds (Maximino et al., 2010; Kalueff et al., 2014a,b).

## Data Availability

The datasets generated for this study are available on request to the corresponding author.

## Author Contributions

MP designed the research. SM and MP secured the funding and supervised the research. YY developed the experimental setup. CS and YY conducted the experiments. All the authors analyzed the data and discussed the results. YY wrote a first draft of the Materials and Methods section and CS wrote a first draft of the manuscript. All the authors reviewed the final draft.

### Conflict of Interest Statement

The authors declare that the research was conducted in the absence of any commercial or financial relationships that could be construed as a potential conflict of interest.

## Supplementary Material

Supplementary Video 1 Front view of the experimental apparatus in the control condition. The robotic platform (without the replica) is visible on the top left side of the video.

Supplementary Video 2 Top view of the experimental apparatus in the control condition. The robotic platform (without the replica) is visible on the left side of the video.

Supplementary Video 3 Front view of the experimental apparatus in the open-loop condition. The robotic platform and the red tiger Oscar fish replica are visible on the left side of the video.

Supplementary Video 4 Top view of the experimental apparatus in the open-loop condition. The robotic platform and the red tiger Oscar fish replica are visible on the left side of the video.

Supplementary Video 5 Front view of the experimental apparatus in the closed-loop condition. The robotic platform and the red tiger Oscar fish replica are visible on the left side of the video.

Supplementary Video 6 Top view of the experimental apparatus in the closed-loop condition. The robotic platform and the red tiger Oscar fish replica are visible on the left side of the video.
